# Cardiovascular and ride time-to-exhaustion effects of an energy drink

**DOI:** 10.1186/1550-2783-11-2

**Published:** 2014-01-22

**Authors:** Michael T Nelson, George R Biltz, Donald R Dengel

**Affiliations:** 1 School of Kinesiology, University of Minnesota, Minneapolis, MN, USA; 2 Department of Pediatrics, University of Minnesota Medical School, Minneapolis, MN, USA

## Abstract

**Background:**

Currently, there are few studies on the cardiovascular and fatigue effects of commercially available energy drinks. This study investigated the effects of Monster energy drink (Monster Beverage Corporation, Corona, California), on resting heart rate (HR), heart rate variability (HRV), ride time-to-exhaustion, peak exercise HR, respiratory exchange ratio (RER), and peak rating of perceived exertion (RPE).

**Methods:**

The study used a double-blind, randomized, placebo controlled, crossover design. After an 8-hr fast, 15 subjects consumed Monster Energy Drink (ED standardized to 2.0 mg * kg^-1^ caffeine) or a flavor-matched placebo preexercise. Resting HR and HRV were determined. After an initial submaximal workload for 30 minutes, subjects completed 10 min at 80% ventilatory threshold (VT) and rode until volitional fatigue at 100% VT.

**Results:**

Resting HR was significantly different (ED: 65+/-10 bpm vs. placebo: 58+/-8 bpm, p = 0.02), but resting HRV was not different between the energy drink and placebo trials. Ride time-to-exhaustion was not significantly different between trials (ED: 45.5+/- 9.8 vs. placebo: 43.8+/-9.3 min, p = 0.62). No difference in peak RPE (ED: 9.1 +/- 0.5 vs. placebo: 9.0 +/- 0.8, p = 1.00) nor peak HR (ED: 177 +/- 11 vs. placebo: 175 +/- 12, p = 0.73) was seen. The RER at 30% of VT was significantly different (ED: 0.94 +/- 0.06 vs. placebo: 0.91 +/- 0.05, p = 0.046), but no difference between the two conditions were seen at the other intensities.

**Conclusion:**

Although preexercise ingestion of the energy drink does increase resting HR there was no alteration in HRV parameters. Ride time-to-exhaustion was not enhanced.

## Background

After a multivitamin, energy drinks (ED) are the most popular dietary supplement in the young adult population [1,2]. Despite their popularity, sparse data exists to support the efficacy and cardiovascular effects, especially in younger adults, which is the target audience [3]. In a small meta-analysis, Shah et al. [4] found that subjects had a 10 mm Hg increase in systolic blood pressure. The main ingredients in most commercially available energy drinks are carbohydrates, B vitamins, caffeine, taurine, herbs, and flavorings.

Caffeine and carbohydrates taken separately have been previously shown to increase exercise duration and capacity [5-9]. A limited number of published studies on preexercise ingestion of energy drinks, however have produced mixed results [10-15]. Some studies showed positive effects such as increased cycling time-trial performance [10], increased bench-press muscle endurance [11], decreased sprint times [13], and increased exercise time at 65-75% of maximum heart rate (HR) on a cycle ergometer [12]. Other studies though [11,14,15], have failed to show any beneficial effect.

Currently there are little data on the cardiovascular effects of energy drinks [16,17]. In addition to caffeine the amino acid taurine, a common energy drink ingredient, is theorized to have potential cardiac effects [18,19]. Bichler and colleagues [20] investigated the combination of caffeine and taurine vs. a placebo and found it actually caused a significant *decline* in heart rate.

The purpose of this study was to investigate a preexercise ingestion of Monster energy drink (Monster Beverage Corporation, Corona, California) on resting HR and HR variability in addition to ride time-to-exhaustion (TTE) in recreationally active young adults. We hypothesize that resting HR and HR variability preexercise will be altered and the ride TTE will be increased after the subjects consume the energy drink (ED standardized to 2.0 mg per kilogram of body mass of caffeine) compared to a taste-matched placebo.

## Methods

### Participants

There were 15 recreationally active subjects (8 male and 7 female). They averaged (mean ± SD) 25.5 ± 4.1 years of age (men 24.1 ± 2.7, women 27.1 ± 5.0), weighed an average of 77.9 ± 18.4 kg (men 86.7 ± 17.6, women 67.9 ± 4.4), had an average body mass index of 25.1 ± 4.0 kg/m^2^ (men 26.6 ± 3.6, women 23.4 ± 3.8), with an average percent body fat of 22.3 ± 8.4% (men 18.0 ± 7.4, women 27.3 ± 6.7), and had an average peak oxygen uptake of 39.5 ± 7.0 ml • kg^–1^ • min^–1^ (men 41.3 ± 3.0, women 37.6 ± 9.7).

Prior to testing, all participants were informed of the study details and procedures including all the potential risks. Participants completed the Physical Activity Readiness Questionnaire which assess their health history [21] and were excluded if they had any significant injury or illness in the previous two weeks. The protocol, informed consent, and related documentation were reviewed by the University of Minnesota Institutional Review Board for approval before the study started and conducted in accordance with their requirements.

### Preliminary testing

Subjects were asked to make three visits to the Laboratory of Integrative Human Physiology (LIHP) on non-consecutive days. The three trips consisted of an initial peak aerobic capacity test and two ride time-to-exhaustion tests, all performed on a stationary electronically braked cycle ergometer (Lode Corival, Groningen, The Netherlands). Subjects were instructed to fast for a minimum of 8 hours previous to all exercise tests, to avoid any caffeine for 48 hours prior, and to not participate in exercise during the previous 24 hours. The 48 hour withdrawal of caffeine was considered adequate given the half-life of caffeine is about 4–6 hours [22]. An overnight fast was done to minimize any effect of the previous meal on respiratory exchange ratio (RER) [23-25]. Subjects were instructed to not change their diet or exercise during the study.

Prior to the first exercise assessment, height and weight were measured using a wall-mounted stadiometer (Ayrton Stadiometer, Model S100, Prior Lake, MN) and digital weight scale (Model 5002, Scale-Tronix Inc., Wheaton, IL). Each measurement was done three times and the mean recorded. Body mass index was calculated as the body weight (kg) divided by height squared (m^2^). Air displacement plethysmography (Bod Pod^®^ Life Measurement Inc., Concord, CA) was used to obtain initial visit body fat percentages. Subjects were instructed to sit still and breathe normally while the body volume measurement was conducted. Thoracic gas volume was estimated according to the methods described by Dempster and Aitkens [26]. Body fat percentage was calculated by computer software using the Siri equation and the collected data [27].

Heart rate was collected prior to exercise to further characterize resting cardiovascular parameters via heart rate variability (HRV) analysis. Participants were prepped for electrode placement for measurement of HR via a 3-lead electrocardiograph (ECG). The ECG (Lead II) was continuously recorded via an automated tonometer (Colin Pilot 7000; Colin Medical Instruments Corp., San Antonio, TX). Participants were asked to pace their breathing at 0.25 Hz (approximately 15 breaths per min) using a computer metronome (Crystal Metronome 1.4.4, MIL software & Matthew Lloyd) cadence. Participants were instructed to lay flat on their backs on a cushioned bed for 10 minutes to ensure that a resting state was attained. After the initial rest period, participants continued to lie relaxed for an additional 10 minutes to record resting ECG measures.

Following resting measures of HR, subjects were fitted with headgear and mouthpiece for collection of expired air by a calibrated open-circuit spirometry metabolic cart (CPX-D, MedGraphics Corporation, St. Paul, MN). Then subjects were fitted with a HR monitor (Polar, Polar Electro Oy, Finland) placed around their chest at the level of the xiphoid process to ensure a quality heart rate signal. Seat and handlebar height were recorded and were replicated for subsequent experimental trials. After warm-up on the bicycle ergometer for 5 minutes at 25 Watts, subjects were asked to complete a progressive resistance exercise test. Subjects rode at a cadence of 60–90 rpm against an increasing resistance of 50 Watts every 2 minutes until volitional exhaustion. Rating of perceived exertion (RPE) was obtained at the end of each stage using the 10-point Borg category scale [28]. All subjects met at least two of the following criteria to be considered a maximal test: 1) increase in VO_2_ between the last 2 stages of less than half the expected increase, 2) RER ≥ 1.10, or 3) RPE ≥ 9 on the Borg 1–10 scale. Analyzed gas samples were used to determine peak aerobic capacity (VO_2 peak_) and the ventilatory threshold (VT) by the D_max_ method [29].

### Experimental design

This study used a randomized, double-blind, placebo controlled, crossover design. Subjects were randomized for preexercise intake with the ED or placebo and received the opposite treatment a minimum of 7 days later (see Table 1 for ingredients). Regular version Monster ED was standardized at 2.0 mg per kilogram of body mass (mg · kgBM^-1^) of caffeine and the placebo was prepared from noncaffeinated diet Mountain Dew and lemon juice by a lab staff member. Both drinks were served in a dark, opaque container and consumed 60 minutes before testing started. The beverage was consumed within a 10-minute period from the time it was received. The mean total beverage volume was 467 ± 109 mL (about one 16 oz can). Resting HR data were obtained as explained above followed by exercise. After a minimum of 7 days from preliminary testing, subjects returned to LIHP for their initial energy drink trial. They observed the same pre-testing criteria with respect to fasting, caffeine, and exercise. All testing was performed in a climate controlled environment between 6:00 to 8:00 am at a minimum of 1 week apart. Participants were informed that they would receive either an energy drink or a taste-matched placebo before experimental testing and a small amount of water (75 mL total) at the 15 minute and 30 minute mark during exercise. Participants were instructed to not discuss the characteristics of the beverages with other participants and were asked at the end of the experimental trial which beverage they received.

**Table 1:** Monster energy drink ingredients

**Ingredient**	**Amount (per kg body mass)**
Carbohydrate	0.65 mg kgBM^-1^
Cafeine	2 mg kgBM^-1^
Taurine	25 mg kgBM^-1^
Pana-ginseng	5 mg kgBM^-1^
Vitamin C	1.5 mg kgBM^-1^
Ribiflavin	0.04 mg kgBM^-1^
Niacin	0.50 mg kgBM^-1^
Vitamin B6	0.05 mg kgBM^-1^
Vitamin B12	0.15 mg kgBM^-1^

### Experimental protocol

After a minimum of 7 days from preliminary testing, subjects returned to LIHP for their initial energy drink trial. They were fitted with headgear and mouthpiece for collection of ventilation, oxygen consumption (VO_2_), carbon dioxide production (VCO_2_), and RER on a breath-by-breath basis. They were also fitted with a HR monitor as described above. After a 5 minute warm up on a bicycle ergometer at 25 Watts, subjects pedaled at a workload corresponding to 30% of their pre-determined VT for 15 minutes, then pedaled at a workload corresponding to 60% of their VT for an additional 15 minutes.

For the ride TTE portion, subjects continued to pedal at 80% of their VT for 10 minutes and then an additional 10 minutes at a workload equal to 100% of VT until volitional fatigue. The total time ride TTE was recorded. Heart rate and RPE were recorded every 2 minutes during exercise. Constant verbal encouragement by the same tester was given to the subjects during each trial to elicit a maximal effort.

The second drink trial was conducted a minimum of 7 days afterwards. Subjects received the opposite assigned preexercise drink from their first exercise trial. The cycle ergometer test protocol and data collection methods remained the same.

### Heart rate variability data analyses

Lead II ECG data for HRV preexercise was collected as described above and were digitally recorded continuously using a desktop computer with WinDaq Pro data collection software (DATAQ Instruments Inc., Akron,OH). The signal was sampled at 500 Hz throughout all testing. The WinDaq Pro software allowed for instantaneous analog to digital conversion of the ECG signal with recordings stored for latter off-line analysis (Kubios Heart Rate Variability software version 2.0 beta 3; Biosignal Analysis and Medical Imaging Group, Kuopio, Finland). Standard time domain parameters [the root mean square of successive differences (RMSSD), the standard deviation of all NN (normal RR) intervals (SDNN) and the percentage of successive NN intervals differing >50 ms (pNN50)] and frequency domain parameters [low frequency power (LF, (0.04 - 0.15 Hz)), high frequency power (HF, (0.15 - 0.4 Hz)) and the ratio of LF/HF] in addition to mean resting HR were calculated. All analysis was performed according to the standards set by the Task Force of the European Society of Cardiology and the North American Society of Pacing and Electrophysiology [30]. The time points from 2 to 8 minutes of the last 10 minute resting period were utilized for calculation of all resting HRV variables. Each 5-minute segment was manually reviewed for ectopic beats or arrhythmias. Segments containing such alterations of normal electrophysiological function were excluded from analysis.

The power spectral density of the RR interval data was calculated using a fast-Fourier transform for the frequency domain parameters. This was based on Welch’s periodogram method to reduce noise in the estimated power spectra with a sampling rate of 4 Hz, and a window width of 256 seconds with an overlap of 50%, corresponding to 128 seconds. Paced breathing was performed to reduce the potential confounding effects of respiratory variation on HRV measures [31].

### Statistical analyses

Beat-by-beat resting HR data was analyzed using Kubios Heart Rate Variability software to obtain the mean HR, time domain, frequency domain, and sample entropy scores for both the supplement and placebo trial. They were compared via a two sample Student’s *t* test. Exercise ride TTE, HR during exercise, and RPE were also analyzed using a two sample Student’s *t* test. Differences were considered significant at p < 0.05. Data are expressed as mean ± SD and were analyzed using SPSS software (version 13.0; SPSS, Inc., Chicago, IL) and Prism^®^ Graphpad Software version 6.0 (Graphpad Software, Inc., San Diego, CA).

## Results

### Preliminary testing

A total of 16 participants completed the study, but one was excluded from the analysis due to heavy exercise prior to testing. Resting HR was significantly higher following the ED than the placebo (ED: 65 ± 10 bpm vs. placebo: 58 ± 8 bpm, p = 0.02). Heart rate variability as calculated via RMSSD, SDNN, pNN50, HF power, LF power, LF/HF ratio, and sample entropy however were not significantly different (see Table 2).

**Table 2:** Comparison of resting heart rate variability parameters under energy drink and placebo conditions

**Parameter**	**Energy drink**	**Placebo**	**p-value**
RMSSD (ms)	76.1 (46.0)	83.7 (54.5)	0.33
SDNN (ms)	94.1 (34.3)	102.0 (51.9)	0.28
pNN50 (%)	38.8 (24.7)	38.8 (21.2)	1.00
LF (ms^2^)	1319 (756)	2295 (2593)	0.12
HF (ms^2^)	4047 (4569)	4235 (5317)	0.79
LF/HF ratio	0.93 (1.15)	0.91 (0.93)	0.90
SampEn	1.33 (0.37)	1.44 (0.37)	0.22

Data are presented as mean (standard deviation). RMSSD - root-mean square differences of successive R-R intervals, SDNN- standard deviation of normal-to-normal intervals, pNN50 percentage of successive NN intervals differing >50 ms, LF - low frequency, HF - high frequency, LF/HF ratio low frequency to high frequency ratio (no units), SampEn - Sample Entropy (no units).

### Experimental testing

**Figure 1:**
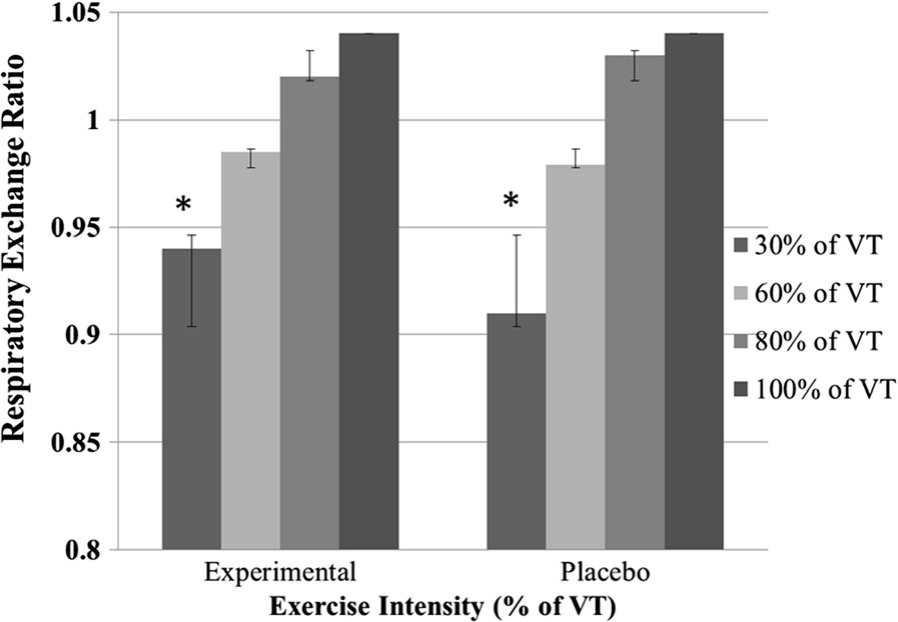
**Respiratory exchange ratio vs. exercise intensity as a percentage of ventilatory threshold (% of VT) for energy drink and placebo conditions.** Values are mean ± standard deviation. Only 30% of VT intensity was different from experimental vs. placebo (*p < 0.046).

Exercise TTE between the ED and the placebo condition was not statistically different between trials (ED: 45.5 ± 9.8 vs. placebo: 43.8 ± 9.3 min p = 0.62). There was no significant difference in peak RPE (ED: 9.1 ± 0.5 vs. placebo: 9.0 ± 0.8, p = 1.00) or peak HR (ED: 177 ± 11 bpm vs. placebo: 175 ± 12 bpm, p = 0.73) during exercise in either the supplement or placebo condition. The RER at 60% VT (ED: 0.99 ± 0.05 vs. placebo: 0.98 ± 0.05, p =0.60), 80% of VT (ED: 1.02 ± 0.07 vs. placebo: 1.03 ± 0.07, p = 0.51), and 100% of VT (ED: 1.04 ± 0.09 vs. placebo: 1.04 ± 0.08, p = 0.62) were not significantly different between the two conditions (Figure 1). The RER at 30% of VT however was significantly higher following the ingestion of ED vs. the placebo (0.94 ± 0.06 vs. 0.91 ± 0.05, p = 0.046). There were no side effects reported from the exercise testing, ED, or placebo. Only one subject dropped out after the initial baseline. At the completion of the experimental trial, six subjects correctly identified the order of ED vs. placebo, four did not, and five were not sure.

## Discussion

This was the first study to investigate preexercise ingestion of the ED Monster in relation to ride TTE and cardiovascular parameters. Cardiovascular parameters at rest did show an increase in HR after consuming the ED, but there were no changes in any HRV parameters. Ride TTE during cycle ergometery testing, peak RPE, and peak HR during exercise were not different between the two conditions. The RER measurements during each intensity were not different between the two conditions, except for the RER at 30% of VT where the placebo condition was lower.

### Exercise effects

The main finding in this study is consistent with data by Candow et al. [14] who conducted a high-intensity run TTE study in young adults (VO_2max_ of 45.5 ± 6.3 ml • kg^–1^ • min^–1^) using a double-blind, crossover, repeated-measures method. They showed no increase in run time or change in RPE with the energy drink Red Bull given preexercise. However, Ivy et al. [10] did see an improvement with preexercise Red Bull. Their study also used a double-blind, randomized, crossover design, but was conducted in athletes with a higher VO_2max_ (54.9 ± 2.3 ml • kg^–1^ • min^–1^) and employed a time trial format. Kazemi et al. [32] demonstrated that Phantom and Dragon energy drinks also significantly increased TTE vs. placebo by 9.3% and 6.5% respectively during a Bruce treadmill test.

### Caffeine

One reason for the lack of increased ride time was possibly the lower dose of caffeine standardized at 2 mg · kgBM^-1^. The recent International Society of Sports Nutrition (ISSN) position stand on energy drinks [33] concluded that although they contain a number of nutrients, the primary ergogenic nutrients appear to be carbohydrate and/or caffeine. The exact mechanism of how caffeine works is still debated, but it is believed to primarily function by acting as an adenosine receptor antagonist, increasing release of free fatty acids, and increasing calcium release and uptake [34]. The track record of positive effects of caffeine is quite good and most studies showed an improvement in exercise capacity in the range of 3–13 mg · kgBM^-1^[9,33,35-40], although Cox et al. [41] did show a decreased time during a time trial performance undertaken at the end of a prolonged cycling bout with a low dose at approximately 1.5 mg · kgBM^-1^. Denadai, et al. [39] used a dose of around 3 mg · kgBM^-1^ and showed that in untrained subjects who exercised below their anaerobic threshold, caffeine increased ride TTE and reduced perceived exertion. Further enhancement in performance does not result when caffeine is consumed in higher dosages (≥ 9 mg · kgBM^-1^).

This study used a standardized dose of 2.0 mg · kgBM^-1^, which is on the lower end for a dose to increase ride TTE. Subjects had to consume the entire ED amount prior to testing, therefore a higher amount may have resulted in gastrointestinal issues due to the increased level of fluid. Subjects were fasted and asked to abstain from caffeine for 48 hours prior to testing, but no other diet controls were applied to make it as applicable to free living subjects as possible.

### Rating of perceived exertion

In the current study, there was no significant difference between peak RPEs when supplementing with an ED or placebo. A meta-analysis in 2005 [42] on caffeine found that it reduced RPE during exercise by 5.6%. Our results are in agreement with Candow et al. [14] and Ivy et al. [10] who did not show any difference in RPE during a high-intensity run time-to-exhaustion and a simulated cycling time trial, respectively.

### Heart rate

Surprisingly, there are little data on the effects of energy drinks on heart rate. No difference was found for peak HR during exercise in this study, but resting HR was higher under the ED condition. Willoughby et al. [16] found HR was unaffected one hour after 50 young adults consumed one 250 ml (8 oz) can of sugar-free Red Bull (approximately 80 mg of caffeine). Steinke et al. [17] however demonstrated that HR was reduced 30 minutes after subjects consumed 75 mg of caffeine. Bichler and colleagues [20] studied a combination of caffeine and taurine, two common ingredients in energy drinks, which resulted in a significant decline in HR.

### Heart rate variability

Heart rate variability may serve as a method to further investigate the cardiac effects of these drinks as it allows quantification of sympathovagal balance [43,44]. Some subjects may be more sensitive to energy drinks resulting in a more sympathetic response, thus altered HRV. In this study, we did not find any difference in time domain, frequency domain, or sample entropy HRV analysis. Since their inception, energy drinks have been suspected of leading to an increased risk of cardiac issues [45]. A recent review on energy drinks [46] regarding safety concluded that there is not enough data currently to allow a definitive dietary recommendation to be made regarding safe levels of ED consumption, and recommended caution. The ISSN Position Stand [33] stated that indiscriminant use of energy drinks, especially if more than one serving per day, may lead to adverse events and harmful side effects.

The only other study on HRV and energy drinks done by Wiklund et al. [47] showed a decreased LF/HF ratio and a tendency to increased HF power (increased vagal modulation). The dose used was high as subjects consumed 3 cans of Red Bull, which represents a dose of 3000 mg of taurine and 240 mg of caffeine after an overnight fast. They also measured RR intervals for the HRV analysis at 30 minutes after the intake of the ED compared to the 60 minute timeframe here. These differences may account for the variance in the results obtained.

As mentioned, the two ingredients in energy drinks that could affect HRV are taurine and caffeine. Taurine has been shown to moderate the flow of cations, especially calcium, across the cell membranes, thus protecting the heart muscle from both high and low concentrations [18,19]. Caffeine is known to increase vagal autonomic nerve activity in resting subjects [48,49]. Ingestion of caffeine preexercise has also been associated with exaggerated vagal withdrawal during post-exercise recovery because of higher baseline level of vagal activity before exercise [49]. However, Rauh et al. [50] did not find any significant differences in respective HRV parameters (HR, RMSSD, SDNN, pNN50, LF, HF and LF/HF) conducted at rest 30, 60, and 90 minutes after 100 and 200 mg caffeine doses were taken and compared to a placebo. They concluded that caffeine at a dose up to 200 mg does not influence HRV [50].

## Conclusion

In conclusion, the results of this present study indicate that consuming Monster ED increases resting HR, but does not increase ride time-to-exhaustion. The ED did not have an impact on parasympathetic and sympathetic balance at rest via HRV analysis. RER was higher after the ED demonstrating a greater reliance on glucose during exercise, but this was only seen at the lowest intensity. The ED did not change the perception of exercise intensity as measured by peak RPE. Future research should compare the effects of regular energy drinks at various caffeine dosages during a ride time-to-exhaustion and a time trial format.

## Abbreviations

ED: Energy drink; BM: Body mass; TTE: Time-to-exhaustion; RPE: Rating of perceived exertion; RER: Respiratory exchange ratio; VO2max: Maximal oxygen consumption; HR: Heart rate; LIHP: Laboratory of Integrative Human Physiology; VO2: Oxygen consumption; CO2: Carbon dioxide; HRV: Heart rate variably; ECG: Electrocardiograph; VO2 peak: Peak aerobic capacity; VT: Ventilatory threshold; SD: Standard deviation; RMSSD: Root mean square of successive differences; SDNN: Standard deviation of all NN (normal RR) intervals; pNN50: Percentage of successive NN intervals differing >50 ms; LF: Low frequency power; HF: High frequency power; LF/HF: Ratio of low frequency to high frequency; ISSN: International Society of Sports Nutrition.

## Competing interest

The authors declare that they have no competing interests.

## Authors’ contributions

MN developed the study design, collected data, conducted statistical analysis, and drafted and submitted the manuscript. DD and GB assisted in the study design, interpretation of data, and critically reviewed the manuscript. All authors read and approved the final manuscript.

## References

[B1] Hoffman JR Faigenbaum AD Ratamess NA Ross R Kang J Tenenbaum G Nutritional supplementation and anabolic steroid use in adolescents Med Sci Sports Exerc 2008 40 1 15 24 18091024 10.1249/mss.0b013e31815a5181

[B2] Froiland K Koszewski W Hingst J Kopecky L Nutritional supplement use among college athletes and their sources of information Int J Sport Nutr Exerc Metab 2004 14 1 104 120 15129934 10.1123/ijsnem.14.1.104

[B3] Clauson KA Shields KM McQueen CE Persad N Safety issues associated with commercially available energy drinks J Am Pharm Assoc (2003) 2008 48 3 e55 e63 quiz e64-7 10.1331/JAPhA.2008.07055 18595815

[B4] Shah S Lacey C Riddock I Impact of energy drinks on electrocardiographic and blood pressure parameters: A meta-analysis of clinical studies [abstract] Circulation 2013 127 AP324

[B5] Noakes TD Lambert EV Lambert MI McArthur PS Myburgh KH Benade AJ Carbohydrate ingestion and muscle glycogen depletion during marathon and ultramarathon racing Eur J Appl Physiol Occup Physiol 1988 57 4 482 489 10.1007/BF00417997 3294002

[B6] Jeukendrup AE Carbohydrate intake during exercise and performance Nutrition 2004 20 7–8 669 677 15212750 10.1016/j.nut.2004.04.017

[B7] Kang J Robertson RJ Goss FL DaSilva SG Visich P Suminski RR Utter AC Denys BC Effect of carbohydrate substrate availability on ratings of perceived exertion during prolonged exercise of moderate intensity Percept Mot Skills 1996 82 2 495 506 10.2466/pms.1996.82.2.495 8724922

[B8] Costill DL Dalsky GP Fink WJ Effects of caffeine ingestion on metabolism and exercise performance Med Sci Sports 1978 10 3 155 158 723503

[B9] Graham TE Spriet LL Performance and metabolic responses to a high caffeine dose during prolonged exercise J Appl Physiol 1991 71 6 2292 2298 1778925 10.1152/jappl.1991.71.6.2292

[B10] Ivy JL Kammer L Ding Z Wang B Bernard JR Liao YH Hwang J Improved cycling time-trial performance after ingestion of a caffeine energy drink Int J Sport Nutr Exerc Metab 2009 19 1 61 78 19403954 10.1123/ijsnem.19.1.61

[B11] Forbes SC Candow DG Little JP Magnus C Chilibeck PD Effect of Red Bull energy drink on repeated Wingate cycle performance and bench-press muscle endurance Int J Sport Nutr Exerc Metab 2007 17 5 433 444 18046053 10.1123/ijsnem.17.5.433

[B12] Alford C Cox H Wescott R The effects of red bull energy drink on human performance and mood Amino Acids 2001 21 2 139 150 10.1007/s007260170021 11665810

[B13] Del Coso J Portillo J Munoz G Abian-Vicen J Gonzalez-Millan C Munoz-Guerra J Caffeine-containing energy drink improves sprint performance during an international rugby sevens competition Amino Acids 2013 44 6 1511 1519 10.1007/s00726-013-1473-5 23462927

[B14] Candow DG Kleisinger AK Grenier S Dorsch KD Effect of sugar-free Red Bull energy drink on high-intensity run time-to-exhaustion in young adults J Strength Cond Res 2009 23 4 1271 1275 10.1519/JSC.0b013e3181a026c2 19528841

[B15] Astorino TA Matera AJ Basinger J Evans M Schurman T Marquez R Effects of red bull energy drink on repeated sprint performance in women athletes Amino Acids 2011 42 5 1803 1808 21461905 10.1007/s00726-011-0900-8

[B16] Willoughby SR Energy drink effects platelet aggregation and endothelial function: A possible link to increased cardiovascular risk Heart Lung Circ 2009 18 S265

[B17] Steinke L Lanfear DE Dhanapal V Kalus JS Effect of “energy drink” consumption on hemodynamic and electrocardiographic parameters in healthy young adults Ann Pharmacother 2009 43 4 596 602 10.1345/aph.1L614 19299320

[B18] Huxtable RJ Physiological actions of taurine Physiol Rev 1992 72 1 101 163 1731369 10.1152/physrev.1992.72.1.101

[B19] Schaffer SW Azuma J Review: myocardial physiological effects of taurine and their significance Adv Exp Med Biol 1992 315 105 120 10.1007/978-1-4615-3436-5_13 1509930

[B20] Bichler A Swenson A Harris MA A combination of caffeine and taurine has no effect on short term memory but induces changes in heart rate and mean arterial blood pressure Amino Acids 2006 31 4 471 476 10.1007/s00726-005-0302-x 16699827

[B21] Adams R Revised physical activity readiness questionnaire Can Fam Physician 1999 45 992 995 1004–5 10216799 PMC2328306

[B22] Graham TE Caffeine and exercise: metabolism, endurance and performance Sports Med 2001 31 11 785 807 10.2165/00007256-200131110-00002 11583104

[B23] Brooks GH Fahey TD Baldwin KDS Exercise Physiology: Human Bioenergetics and its Applications 2005 Boston: McGraw-Hill

[B24] Wilmore JH Costill DL Physiology of Sport and Exercise 2007 4 Champaign, Illinois: Human Kinetics Publishers

[B25] Miller SL Maresh CM Armstrong LE Ebbeling CB Lennon S Rodriguez NR Metabolic response to provision of mixed protein-carbohydrate supplementation during endurance exercise Int J Sport Nutr Exerc Metab 2002 12 4 384 397 12500983 10.1123/ijsnem.12.4.384

[B26] Dempster P Aitkens S A new air displacement method for the determination of human body composition Med Sci Sports Exerc 1995 27 12 1692 1697 8614327

[B27] Siri WE Body composition from fluid spaces and density: analysis of methods. 1961 Nutrition 1993 9 5 480 491 8286893

[B28] Borg GA Psychophysical bases of perceived exertion Med Sci Sports Exerc 1982 14 5 377 381 7154893

[B29] Cheng B Kuipers H Snyder AC Keizer HA Jeukendrup A Hesselink M A new approach for the determination of ventilatory and lactate thresholds Int J Sports Med 1992 13 7 518 522 10.1055/s-2007-1021309 1459746

[B30] Task Force of the European Society of Cardiology and the North American Society of Pacing and Electrophysiology Heart rate variability: standards of measurement, physiological interpretation and clinical use Circulation 1996 93 5 1043 1065 10.1161/01.CIR.93.5.1043 8598068

[B31] Katona PG Jih F Respiratory sinus arrhythmia: noninvasive measure of parasympathetic cardiac control J Appl Physiol 1975 39 5 801 805 1184518 10.1152/jappl.1975.39.5.801

[B32] Kazemi F Gaeini A Kordi M Rahnama N The acute effects of two energy drinks on endurance performance in female athlete students Sport Sci Health 2009 5 2 55 60 10.1007/s11332-009-0077-7

[B33] Campbell B Wilborn C La Bounty P Taylor L Nelson MT Greenwood M Ziegenfuss TN Lopez HL Hoffman JR Stout JR Schmitz S Collins R Kalman DS Antonio J Kreider RB International Society of Sports Nutrition position stand: energy drinks J Int Soc Sports Nutr 2013 10 1 1 2783 10-1 10.1186/1550-2783-10-1 23281794 PMC3538552

[B34] Davis JK Green JM Caffeine and anaerobic performance: ergogenic value and mechanisms of action Sports Med 2009 39 10 813 832 10.2165/11317770-000000000-00000 19757860

[B35] Kovacs EM Stegen JHCH Brouns F Effect of caffeinated drinks on substrate metabolism, caffeine excretion, and performance J Appl Physiol 1998 85 2 709 715 9688750 10.1152/jappl.1998.85.2.709

[B36] Spriet LL MacLean DA Dyck DJ Hultman E Cederblad G Graham TE Caffeine ingestion and muscle metabolism during prolonged exercise in humans Am J Physiol 1992 262 6 Pt 1 E891 E898 1616022 10.1152/ajpendo.1992.262.6.E891

[B37] Graham TE Spriet LL Metabolic, catecholamine, and exercise performance responses to various doses of caffeine J Appl Physiol 1995 78 3 867 874 7775331 10.1152/jappl.1995.78.3.867

[B38] Pasman WJ van Baak MA Jeukendrup AE de Haan A The effect of different dosages of caffeine on endurance performance time Int J Sports Med 1995 16 4 225 230 10.1055/s-2007-972996 7657415

[B39] Denadai BS Denadai ML Effects of caffeine on time to exhaustion in exercise performed below and above the anaerobic threshold Braz J Med Biol Res 1998 31 4 581 585 9698813 10.1590/s0100-879x1998000400017

[B40] Burke LM Caffeine and sports performance Appl Physiol Nutr Metab 2008 33 6 1319 1334 10.1139/H08-130 19088794

[B41] Cox GR Desbrow B Montgomery PG Anderson ME Bruce CR Macrides TA Martin DT Moquin A Roberts A Hawley JA Burke LM Effect of different protocols of caffeine intake on metabolism and endurance performance J Appl Physiol 2002 93 3 990 999 12183495 10.1152/japplphysiol.00249.2002

[B42] Doherty M Smith PM Effects of caffeine ingestion on rating of perceived exertion during and after exercise: a meta-analysis Scand J Med Sci Sports 2005 15 2 69 78 10.1111/j.1600-0838.2005.00445.x 15773860

[B43] Montano N Ruscone TG Porta A Lombardi F Pagani M Malliani A Power spectrum analysis of heart rate variability to assess the changes in sympathovagal balance during graded orthostatic tilt Circulation 1994 90 4 1826 1831 10.1161/01.CIR.90.4.1826 7923668

[B44] Eckberg DL Sympathovagal balance: a critical appraisal Circulation 1997 96 9 3224 3232 10.1161/01.CIR.96.9.3224 9386196

[B45] Finnegan D The health effects of stimulant drinks Br Nutr Found Nutr Bull 2003 28 147 155 10.1046/j.1467-3010.2003.00345.x

[B46] Burrows T Pursey K Neve M Stanwell P What are the health implications associated with the consumption of energy drinks? A systematic review Nutr Rev 2013 71 3 135 148 10.1111/nure.12005 23452281

[B47] Wiklund U Karlsson M Ostrom M Messner T Influence of energy drinks and alcohol on post-exercise heart rate recovery and heart rate variability Clin Physiol Funct Imaging 2009 29 1 74 80 10.1111/j.1475-097X.2008.00837.x 19016812

[B48] Hibino G Moritani T Kawada T Fushiki T Caffeine enhances modulation of parasympathetic nerve activity in humans: quantification using power spectral analysis J Nutr 1997 127 7 1422 1427 9202101 10.1093/jn/127.7.1422

[B49] Yeragani VK Krishnan S Engels HJ Gretebeck R Effects of caffeine on linear and nonlinear measures of heart rate variability before and after exercise Depress Anxiety 2005 21 3 130 134 10.1002/da.20061 15965989

[B50] Rauh R Burkert M Siepmann M Mueck-Weymann M Acute effects of caffeine on heart rate variability in habitual caffeine consumers Clin Physiol Funct Imaging 2006 26 3 163 166 10.1111/j.1475-097X.2006.00663.x 16640511

